# Intravenous bisphosphonate-related osteonecrosis of the jaws: Influence of coadjuvant antineoplastic treatment and study of buccodental condition

**DOI:** 10.4317/medoral.18604

**Published:** 2012-12-10

**Authors:** Maria Margaix-Muñoz, José Bagán, Rafael Poveda-Roda

**Affiliations:** 1 Associate Professor of Oral Medicine. Oral Medicine Unit. Department of Stomatology. University of Valencia; 2 Chairman of Oral Medicine. Oral Medicine Unit. Department of Stomatology. University of Valencia. Head of the Department of Stomatology and Maxillofacial Surgery. Valencia University General Hospital; 3 Staff physician. Department of Stomatology and Maxillofacial Surgery. Valencia University General Hospital

## Abstract

Objectives: To determine whether coadjuvant antineoplastic treatment can influence the number and size of bone exposures among patients with intravenous bisphosphonate-related osteonecrosis of the jaws (iBRONJ), and to analyze the buccodental condition of these patients. Material and methods: The study sample comprised 67 patients with iBRONJ, 53 patients without iBRONJ receiving treatment with intravenous bisphosphonates, and 36 healthy subjects. In all three groups, measurements were made of the CAO index and of resting whole saliva and stimulated whole saliva. In the patients with iBRONJ, the size (cm) and number of bone exposures were recorded. The data obtained were subjected to analysis of variance (ANOVA), the Mann-Whitney U-test, and multivariate logistic regression analysis. Results: A total of 57.6% of the patients presented single bone exposure, 25.4% presented two, and 17% more than two exposures. The mean exposure size was 2.3±1.9 cm. Neither the bivariate analysis nor the multivariate multiple regression analysis found coadjuvant antineoplastic treatment to exert a statistically significant effect upon the number and size of bone exposures. On the other hand, there were statistically significant differences among the three study groups in relation to the CAO index (p=0.02) and the number of missing teeth (p=0.00). The resting whole saliva and stimulated whole saliva levels were similar in the three groups, though the patients with osteonecrosis of the jaws showed comparatively lower SWS levels. Conclusions: Coadjuvant antineoplastic treatment alone appears to exert no influence upon the size and number of bone exposures in iBRONJ. The patients with this disease show a higher CAO index and a larger number of missing teeth.

** Key words:**Osteonecrosis of the jaws, bisphosphonates, bone exposure, CAO index, resting whole saliva, stimulated whole saliva.

## Introduction

Osteonecrosis of the jaws is a form of chronic and slow-evolving osteomyelitis with no tendency towards spontaneous healing. The most widely known clinical form is osteoradionecrosis, a form of osteomyelitis associated to radiotherapy (1). Since the year 2003, a new form of osteonecrosis has been described, referred to as bisphosphonate-related osteonecrosis of the jaws (BRONJ), which is defined as the presence of necrotic bone exposed within the oral cavity for over 6-8 weeks in patients receiving or who have received treatment with bisphosphonates (BPs), and who have not undergone radiotherapy in the cervicofacial region (2,3). Instead of necrotic bone exposure, the disease in some cases is characterized by mucosal ulceration and symptoms - usually pain and suppuration, and even orosinusal communications and pathological fractures. This condition is also regarded as bisphosphonate-related osteonecrosis of the jaws (4,5).

The pathogenesis of BRONJ is not fully understood. The most widely accepted hypothesis refers to the existence of an alteration in physiological bone remodeling secondary to intense inhibition of the osteoclasts. Bisphosphonates inhibit osteoclast reabsorption activity by blocking 3-hydroxy-3-methylglutaryl coenzyme A (HMG-CoA), the enzyme in charge of osteoclast protein prenylation (6). The appearance of cases of BRONJ in patients receiving treatment with denosumab (7), an anti-RANK ligand monoclonal antibody, confirms the theory that osteoclast inhibition is the triggering event underlying BRONJ (8). The BPs have also been attributed with certain antiangiogenic and antitumor effects (9).

BRONJ has very characteristic clinical manifestations that allow it to be easily identified from first contact with the patient (10).

Major risk factors for BRONJ are the potency and duration of intravenous BP treatment, particularly with zoledronic acid, and dentoalveolar surgery – fundamentally extractions (11). Other treatments commonly administered to cancer patients, such as corticosteroids, immunosuppressors and hormone therapy, may also increase the risk of suffering BRONJ (12). It has been suggested that certain polymorphisms of the CYP2C8 gene could induce a degree of genetic susceptibility towards BRONJ, though this finding has not been confirmed by other studies (13).

 The great majority of authors and expert panels agree on the importance of preventing BRONJ through the establishment and maintenance of adequate buccodental health (2,3,14,15). A rapid and simple method for assessing buccodental health is the CAO index, described by Klein, Palmer and Knutson (16), and adopted by the World Health Organization (WHO) for the conduction of oral health surveys, with the measurement of present and past caries in an individual or population.

**Figure 1:**
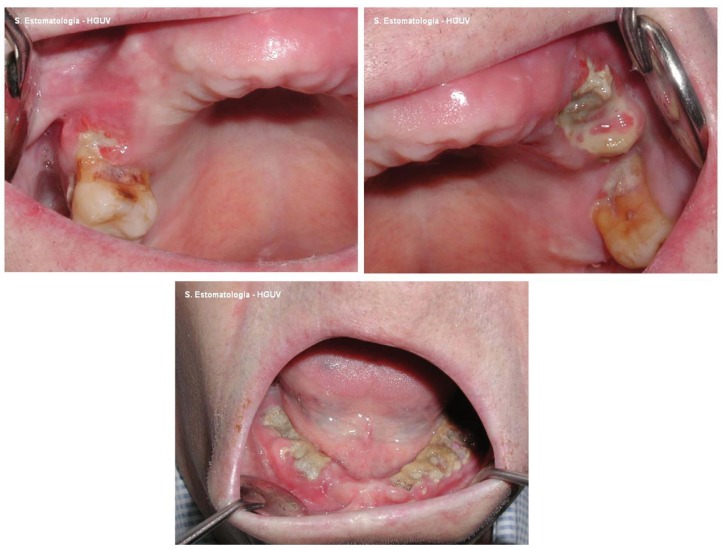
Images of one same patient with multiple bone exposure sites, corresponding to intravenous bisphosphonate-related osteonecrosis of the jaws.

Considering the natural tendency of intravenous BRONJ lesions to progress and spread over time (Fig. 1), the first objective of the present study was the evaluation of whether coadjuvant antineoplastic treatment such as corticosteroids, thalidomide, interferon and hormones can influence the number and size of bone exposures. On the other hand, we have only found one publication comparing the buccodental conditions of patients receiving treatment with intravenous bisphosphonates versus those of patients who have already developed osteonecrosis of the jaws (17). Our second objective therefore has been the analysis and comparison of buccodental health and of the resting whole saliva and stimulated whole saliva levels in a group of patients with iBRONJ versus a group of patients administered BP via the intravenous route but who do not present BRONJ, and un control group of healthy patients.

## Material and Methods

All patients were seen in the Department of Stomatology and Maxillofacial Surgery (Valencia University General Hospital, Valencia, Spain) during the period between March 2006 and July 2009, in collaboration with the Departments of Hematological Oncology of Valencia University Clinic Hospital, La Fe University Hospital and Valencia University General Hospital (Valencia, Spain). Informed consent was obtained in all cases, and the study was approved by the Clinical Research Ethics Committee of Valencia University General Hospital.

The study sample consisted of 156 patients, 105 women (67.3%) and 51 men (32.7%), with a mean age (± SD) of 62.3±10.9 years. Three groups were established:

- Group A: 67 patients administered intravenous bisphosphonates and who developed iBRONJ: 39 women and 28 men, with a mean age of 63.7 ± 11.6 years.

- Group B: 53 patients administered intravenous bisphosphonates but without BRONJ: 37 women and 16 men, with a mean age of 60.3±10.9 years.

- Group C: 36 healthy controls matched for age and gender to the other two groups.

Data were collected referred to the background malignancy, iBRONJ and buccodental condition. For the clinical exploration we used an intraoral mirror and exploratory probe. The diagnosis of BRONJ was based on the corresponding clinical and radiological criteria (2,3,12). Samples of resting whole saliva (RWS) and stimulated whole saliva (SWS) were collected. Saliva measurement was not possible in 28 patients in group A. In the remaining 128 patients, saliva samples were collected as described below.

Sampling was carried out early in the morning in the dental clinic, under fasting conditions or after at least two hours without oral ingestion of any kind. Before collecting the saliva sample, the patients were instructed to rinse the oral cavity with desalinized water and then expel the water. The RWS sample was collected first, instructing the patients to expel the saliva accumulated in the floor of the mouth every 60 seconds over a period of 5 minutes. Emphasis was placed on the importance of not swallowing the saliva produced. The saliva was collected through a glass funnel in a 15-ml, millimetered Falcon-type tube (VWR Internacional Eurolab, S.L., Barcelona, Spain). Two minutes after completing collection of the resting sample, the SWS sample was collected. Paraffin blocks measuring 1.5 x 1 x 0.5 cm in size were used to this effect (CRT Paraffin Vivadent®. Ivoclar Vivadent AG, Liechtenstein), instructing the patients to keep a uniform chewing rhythm throughout the procedure (average of 70 mastications/minute), and to avoid swallowing the saliva produced. In contrast to the sampling of resting saliva, the saliva produced in the first two minutes was discarded, with collection of the saliva generated in the subsequent 5 minutes, using the same system as in the case of RWS sampling. The amount of saliva obtained under both resting and stimulated conditions was then measured, expressed as ml of saliva/5 minutes.

The predominant background malignancy in groups A and B was multiple myeloma (45.8%), followed by breast (41.5%), prostate (9.3%), lung (1.7%), kidney (0.8%) and bladder cancer (0.8%). The most frequently administered bisphosphonate was zoledronic acid (97.5%). The patients in group B received only zoledronic acid, while in group A, 66.2% of the patients received zoledronic acid, 31.8% received zoledronic acid associated to pamidronate, and 2% received only pamidronate ( Table 1).

**Table 1:**
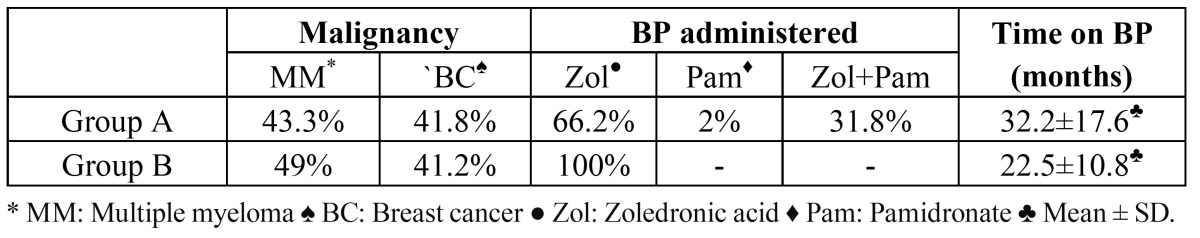
Comparison between groups A and B referred to type of primary neoplasm, type of bisphosphonate (BP) administered, and duration of treatment.

The data obtained were analyzed using the SPSS version 15.0 statistical package (SPSS Inc., Chicago IL, USA). A descriptive study was made, calculating the mean, standard deviation and frequency distribution. The comparative study in turn was based on analysis of variance (ANOVA), the Mann-Whitney U-test, and multivariate logistic regression analysis. Statistical significance was considered for p<0.05.

## Results

-Number and size of bone exposures

**Figure 2:**
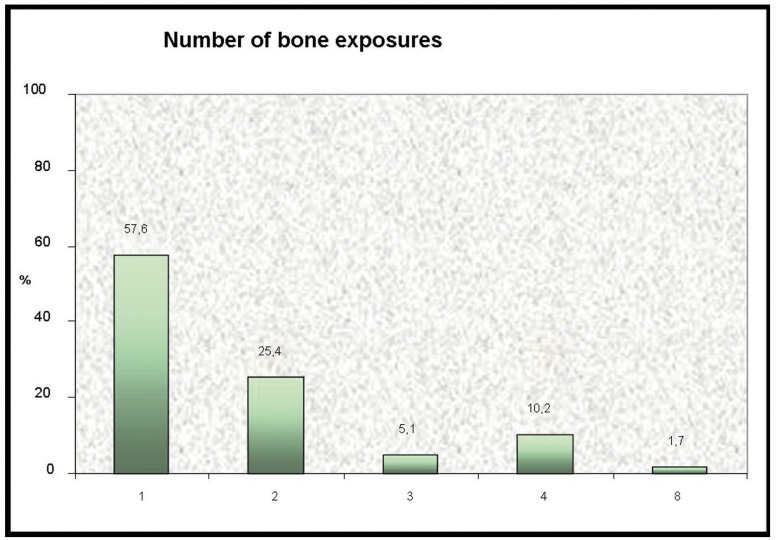
Number of bone exposure sites recorded in the patients with intravenous bisphosphonate-related osteonecrosis of the jaws.

A total of 57.6% of the patients presented single bone exposure, 25.4% presented two, 5.1% three, 10.2% four, and 1.7% presented 8 exposures (Fig. 2). The mean exposure size was 2.3±1.9 cm (median 2 cm). In the bivariate analysis, none of the coadjuvant antineoplastic treatments considered (corticosteroids, thalidomide, interferon and hormones) were significantly related to either the size or number of exposures ( Table 2). In turn, in none of the logistic regression analysis models was coadjuvant antineoplastic treatment able to explain and predict the total size or number of exposures.

**Table 2:**
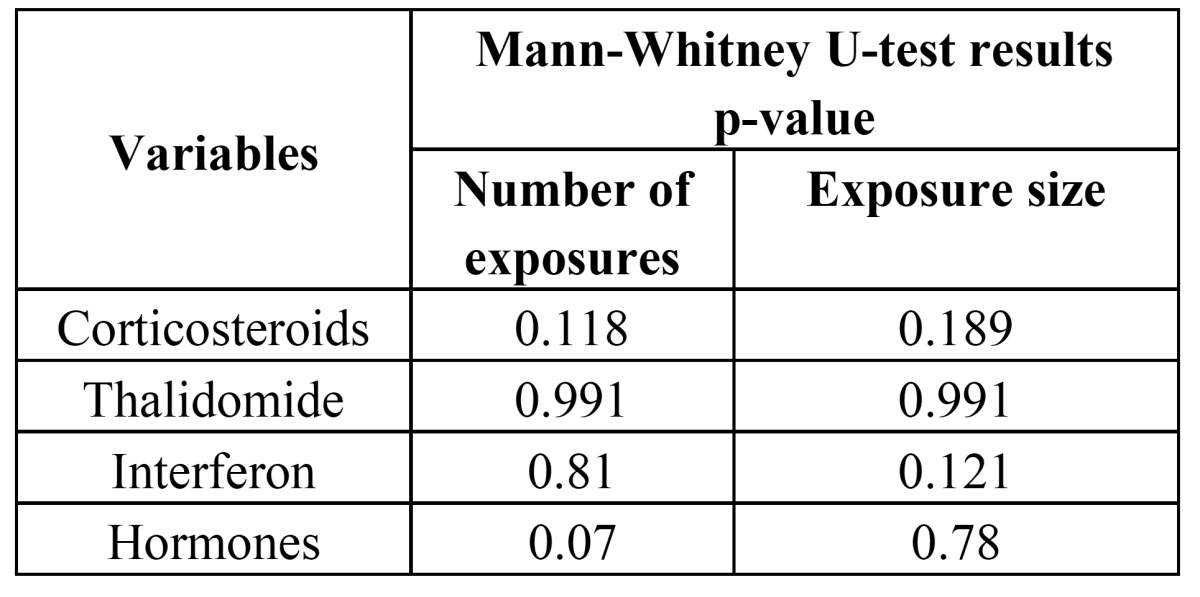
Results of the bivariate analysis (Mann-Whitney U-test) of the number and size of bone exposures.

-CAO index

The mean CAO index (ICAO) for the total sample was 14.7. In group A (BRONJ) the mean ICAO was 16.8, versus 12.5 in group B (without BRONJ) and 13.4 in group C (control). The difference in ICAO distribution in the three groups was statistically sig-nificant (p=0.02).

On analyzing ICAO more in detail, the global mean number of caried teeth was found to be 1.6, with individualized values of 1.7, 1.8 and 0.8 for groups A, B and C, respectively. These differences were not statistically significant (p>0.05). The global mean number of missing teeth was 10.7. Statistically significant differences were observed in the mean number of missing teeth among the three study groups: 14.3 in group A, 6.8 in group B and 9.1 among the controls (group C) (p=0.00). Lastly, the global mean number of filled teeth was 3.4. No statistically significant differences were observed in the mean number of filled teeth among the groups: 3.1 in group A, 3.8 in group B and 3.4 in group C (p>0.05). The results referred to the CAO index and its variables are summarized in  Table 3.

**Table 3:**
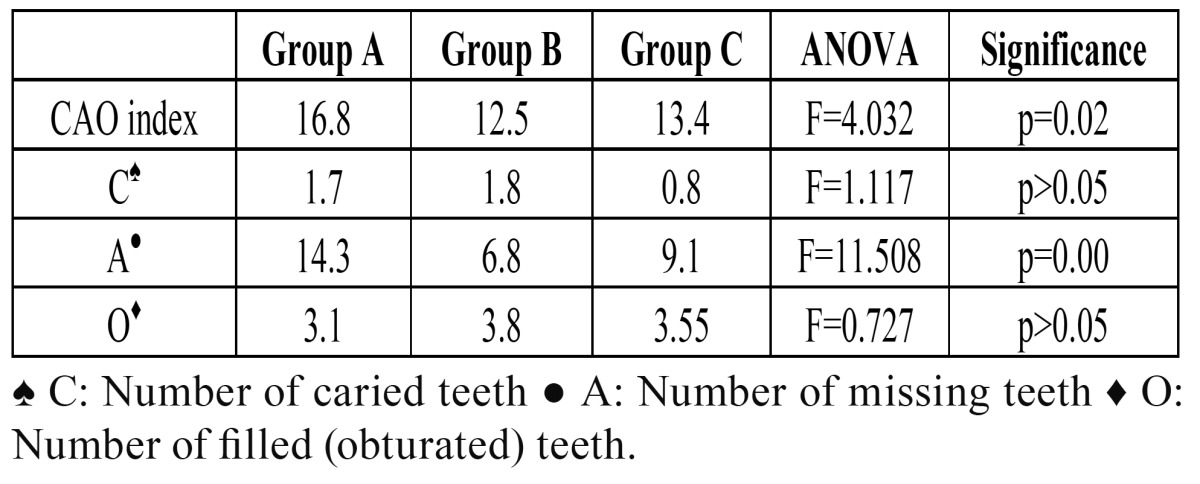
Mean values in each study group corresponding to the CAO index and its variables.

-Resting whole saliva (RWS) and stimulated whole saliva (SWS)

The global mean amount of RWS obtained in the 128 patients was 1.1 ml saliva/5 minutes. In group A (BRONJ) the mean amount was 1.3 ml/5 minutes, versus 1.1 ml/5 minutes in group B (without BRONJ), and 1 ml/5 minutes in group C (controls). In turn, the global mean amount of SWS obtained was 3.1 ml saliva/5 minutes. In group A the mean amount was 2.7 ml/5 minutes, versus 3.2 ml/5 minutes in group B, and 3.3 ml/5 minutes in group C. No statistically significant differences were found on comparing the mean amounts of RWS and SWS obtained in each group (p>0.05) ( Table 4).

**Table 4:**
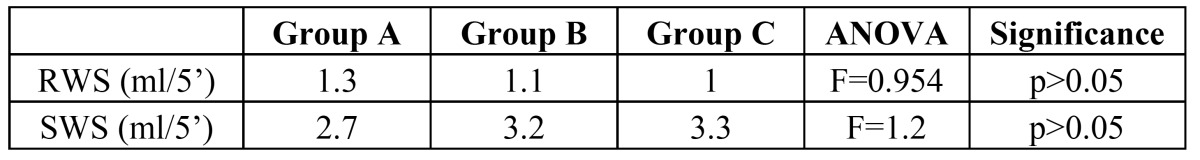
Mean values in each study group corresponding to resting whole saliva (RWS) and stimulated whole saliva (SWS).

## Discussion

The two main disorders associated to prolonged bisphosphonate therapy are BRONJ and pathological femoral fracture (18). The apparently exclusive location of BRONJ in the oral cavity, and specifically in jaw bone, appears to be related to the fact that this bone has a high turnover rate, which favors the accumulation of large amounts of bisphosphonates (19). The main risk factors for BRONJ are prolonged intravenous bisphosphonate therapy involving high-potency drugs, dentoalveolar surgical procedures, poorly fitting dental prostheses and intraoral trauma (20). Other factors that have also been related to BRONJ are chemotherapy, antiangiogenic drugs such as thalidomide, diabetes mellitus, corticosteroids, genetic susceptibility and deficient oral hygiene (21).

The different published series on osteonecrosis of the jaws generally do not describe the number or size of the bone exposure sites. At most, mention is made of the location of exposure in the upper or lower jaw, or both (22,23). We have found only three studies, published by Bagán et al. (1,10,11), in which express mention is made of the number of areas of exposed bone and their global mean size.

More than one-half of our patients (57.6%) presented a single bone exposure site, while 25.4% had two and 17% presented more than two exposures. This latter subgroup included patients with up to 8 iBRONJ exposure sites. According to Thumbigere-Math et al. (24), 80% of all spontaneously manifesting iBRONJ lesions measure 1 cm or less in diameter, and a little over one-half of the lesions appearing after a dental procedure (56.3%) have these same dimensions (i.e., 1 cm or less). In our series, 50% of the patients presented a lesion size of between 1-3 cm, though there were two individuals with an unusually large lesion (7 cm), and one patient with an extreme lesion size of 10 cm. It should be underscored that on the basis of our findings, coadjuvant antineoplastic treatment alone exerted no influence upon the size and number of osteonecrosis areas in the context of iBRONJ.

A greater CAO index and a larger number of missing teeth were found in the patients with iBRONJ (p<0.05). The study published by Carmagnola et al. (17), which did not include a control group, reported no statistically significant differences in either the CAO index or in residual periodontal support between 20 patients with iBRONJ and 19 patients subjected to intravenous bisphosphonates without BRONJ. In that same publication, 50% of the patients with iBRONJ had a history of tooth extraction, versus 26.3% of the subjects without BRONJ. In our series, 73.1% of the patients in group A (BRONJ) had undergone tooth extraction, versus only 9.4% of those in group B (without BRONJ). These figures are similar to those recorded in the first published series on osteonecrosis of the jaws, in which 77% of the patients had undergone dental extraction (23). Likewise, Boonyapakorn et al. (25), in a study of 80 patients with BRONJ, found 77% of them to have a history of extraction. We found no statistically significant differences among the three groups in terms of the number of caried teeth or the number of filled teeth (p>0.05), though both caried and filled teeth were more prevalent in group B.

The RWS levels were practically the same in all three groups (p>0.05). Certain differences were observed in the case of SWS, with lesser levels among the patients with BRONJ – though statistical significance was not reached.

At the time of intraoral exploration and collection of the saliva samples, 94% of the patients with BRONJ were receiving chemotherapy – a percentage that dropped to 68% in the case of group B (without BRONJ). These data could partially account for the observed differences among the study groups as regards buccodental condition. The fact that the patients with BRONJ had a longer duration of treatment with bisphosphonates, and the concomitant administration of chemotherapy, could have influenced the existence of oral cavity disorders such as diminished salivary gland function, increased susceptibility to fungal, viral and bacterial infections, and pro-pathogenic changes in the oral microflora (26,27). The greater number of missing teeth among the patients in group A might be related to the above.

A growing number of studies underscore the beneficial effects of oral health prevention and maintenance strategies in patients programmed for or who are already receiving intravenous bisphosphonate therapy (28,29). In relation to BRONJ, we feel that studies addressing the buccodental health of these patients are required.

## Conclusion

Coadjuvant antineoplastic treatment alone appears to exert no influence upon the size and number of exposed bone sites in the context of i.v. BRONJ – the patients with this disease in turn showing a comparatively greater CAO index and a larger number of missing teeth.
